# International Comparison of Social Support Policies on Long-Term Care in Workplaces in Aging Societies

**DOI:** 10.3390/ijerph19063284

**Published:** 2022-03-10

**Authors:** Koji Kanda, Hirofumi Sakurazawa, Takahiko Yoshida

**Affiliations:** 1 Department of Social Medicine, Asahikawa Medical University, Asahikawa 078-8510, Japan; tyoshida@asahikawa-med.ac.jp; 2 Paragon, LLC, Tokyo 107-0062, Japan; sakura@pro-sangyoui.com

**Keywords:** Europe, Japan, long-term care (LTC), North America, occupational health, work–life balance, working-age population

## Abstract

A decrease in the working-age population in aging societies causes a shortage of employees in workplaces due to long-term care (LTC) leave for family and relatives as well as longer working hours or overwork among those remaining in the workplace. We collected and analyzed literature and guidelines regarding social-support policies on LTC in workplaces in seven countries (Canada, France, Germany, Japan, Sweden, the UK, and the USA) to propose an effective way of occupational health support for those in need. Our analysis indicated the existence of a system that incorporates the public-assistance mechanism of providing unused paid leave to those in need. Additionally, recipients of informal care provided by employees tended to expand to non-family members under the current occupational health system. On the other hand, the health management of employees as informal caregivers remained neglected. Likewise, salary compensation and financial support for LTC-related leave need to be improved. In order to monitor and evaluate the progress and achievement of current legal occupational health systems and programs related to the social support of LTC among employees, the available national and/or state-based quantitative data should be comparable at the international level.

## 1. Introduction

The demand for long-term care (LTC) has been increasing recently in aging societies. A proportion of the total population aged 65 and over among the countries of the Organisation for Economic Co-operation and Development (OECD) has increased from 13.0% to 17.5% over the last two decades since 2000 [1]. The number of people receiving LTC at home among 15 OECD countries in 2010–2019 has also increased by 72% [1]. On the other hand, the working-age population has declined from 66.2% to 64.8% in 2000–2020, indicating that the old-age dependency ratio or people aged 65 or above relative to those aged 15–64 (ODR) has risen from 19.4% to 27.0% [1]. If this trend continues, the ODR is estimated to reach 38.7% in 2040 and 45.2% in 2060, and it is already close to 50% in some countries such as Japan (48.9%) [1].

Under such circumstances, the demand for LTC by the working-age population as informal caregivers is likely to increase further in the future. Particularly, informal caregivers are preferably accepted in many countries and regions, possibly due to lack of accessible formal LTC facilities, poor quality of LTC, and traditional model of intergenerational and familial relations [2,3,4]. Thus, LTC policies and legislation have been developed and amended according to the latest situation of LTC in each country. In Europe, regionwide conferences explored challenges and good practices in informal long-term care provision based on the latest LTC policies and legislation [5]. In Japan, the Child and Family Care Leave Law has been revised periodically to promote support for balancing work and family care [6]. Nonetheless, necessary supports for employees engaged in LTC as informal caregivers have not been well directed as part of occupational health. A recent interview among working caregivers indicated that there was a lack of mutual understanding of LTC between employers and caregivers, resulting in inadequate workplace supports, which are essential to satisfy the needs of working caregivers in different stages of LTC [7]. Providing informal care while being employed requires a balance between the care-dependent person and the workplace responsibilities of the caregiver [8]. In fact, a large number of employees engaged in informal face difficulties in managing their daily work and family and relative care, including the limitation of their working hours or drop-out of their current work [3,4,9,10,11,12,13,14,15,16]. Conversely, work performance can also be affected by caregiving, resulting in fewer promotions and a less-demanding job in the workplace [17]. It is frequently reported that women, in particular, seem to be more affected by the balance between both LTC and work restrictions [2,10,11,13,15,18]. They are often at risk of economic deprivation [13,15,19]. The poor in particular tend to be reluctant to use formal care for financial reasons [20]. The hidden cost of informal care is not well discussed, and a report indicates that it is twice as expensive as formal care [20]. A large number of informal caregivers also suffer from unfavorable physical and/or mental health conditions due to their responsibilities of caregiving and other life events such as job and/or household work [15,16,17,21]. Experiencing work interference or a change in work status due to caregiving is also associated with greater emotional stress [22]. Hence, the need for respite, wishing for a break, and balancing work and care have been discussed elsewhere [8,20,23,24,25,26,27]. On the other hand, generally low awareness of the legal LTC support system prevents employees from making proper use of existing supports [2,12,13,15,28]. There is also a disparity in the use of the LTC support system depending on the region of residence. Informal caregivers living in rural areas are reported to have more limited access to the company’s system of care than those living in urban areas [29]. Even if they are able to take leave, there are cases where they are reluctant to use the system itself due to company culture or social stigma [13]. However, supportive organizations were more likely to ensure caregivers’ work performance and to maintain low stress at work [26,30]. On the national level, gaps between existing needs and available supports are reported [27]. Additionally, countries providing extensive health and LTC support systems are more likely to report minimal negative effects on work than countries with limited support [17].

As described above, there are still many improvements that need to be made in the system for the protection of workers in the occupational health field who are caring for family members or relatives, and there is ample room to consider how to continue to effectively support them as informal caregivers. Therefore, we compared and examined the current occupational health policies and regulations regarding the LTC support for employees, and proposed how both the government and companies should provide a better working environment for those in need of LTC. Particularly, we evaluated the current policy and regulation of LTC among workforces in selected countries using the existing literature to address how we formulate a better occupational health environment for them.

## 2. Materials and Methods

**Figure 1:**
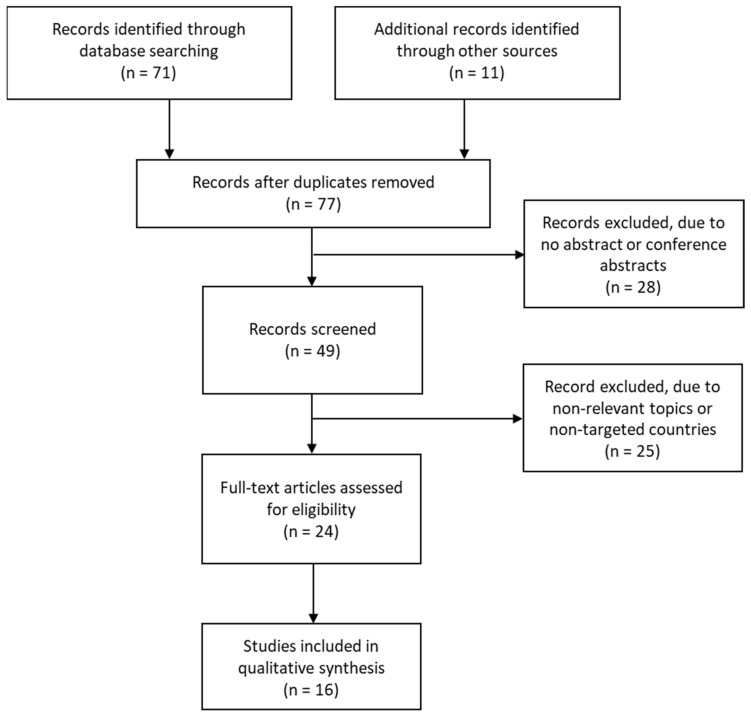
Document-screening process.

This study examined the social support for workers in need of LTC for family members and its current status in different countries, using existing policy documents and data. Seven OECD-member countries were selected: Canada, France, Germany, Japan, Sweden, the United Kingdom, (UK), and the United States of America (USA). The policy documents that related to occupational health and LTC were obtained from each government’s website and academic journals in search engines, such as Pubmed, Scopus, and Europe PMC. The following keywords were selected to identify the articles: informal caregiver, long-term care, policy, workplace. Moreover, we utilized the latest edition of the Overseas Situation Report published by the Ministry of Health, Labour and Welfare of Japan, which overviewed the health and labor policies, and their recent trends, in selected countries, including the above seven nations except for Japan [31]. Then, the selected documents were screened according to the Preferred Reporting Items for Systematic reviews and Meta-Analyses (PRISMA) statement [32]. As a result, a total of 16 articles were selected for the analysis (Figure 1). On the other hand, as quantitative figures, we extracted related data on occupational health and LTC from the websites of UN agencies and international organizations such as the World Health Organization (WHO), International Labour Organization (ILO), and OECD to depict the current situation of social support systems.

The analysis was based on the following two topics. First, as “Social support for work–life balance focusing on LTC, based on occupational health standards and LTC-related laws and guidelines”, we summarized the basic rules and regulations related to occupational health in each country and their history in reaching the currently available LTC support in terms of the legal system. The second topic is “A detail of LTC support systems for employees who take care of family members, including shorter working hours, paid or unpaid leave, and financial compensation”. We overviewed each country’s specific rules to protect employees in various situations towards LTC for their family and relatives, such as shorter working hours and restrictions on late-night work as well as LTC-leave systems and salary compensation for leave. The extent to which these systems are recognized and utilized was assessed through available official data published in each country.

## 3. Results

### 3.1. Basic Information and Occupational Health Standards in Each Country

Table 1 shows the demographic information and occupational health standards of seven countries. The working-age population was found to decline in all countries between 2005–2020. Accordingly, the old-age dependency ratio, the number of people in 15–64 years of age supporting for a person aged 65 years and above, exceeded 0.25 in 2020. Particularly, Japan has almost reached 0.5, meaning that one elderly person is supported by only two working-age people. In all countries, it is projected that less than three persons will support one elderly person by 2040. Now, life expectancy at birth is over 80 years old except in the USA, while the gap between life expectancy and healthy life expectancy now extends to more than 10 years. This indicates that those who are over 70 years of age find it more difficult to live independently and are more likely to receive any sort of care in their daily life.

Table 2 shows the working conditions and trends for workers in the seven countries of study [31,33,34,35,36,37]. The legal working hours are 8 h per day in Canada, Germany, and Japan, and 40 h per week in Canada, Japan, and Sweden. The USA also has a 40 h week, but only for federal employees and in several states. The OECD statistics showed that the average weekly working hours did not exceed 40 h in any country, although France slightly exceeded the legal working hours. On the other hand, in Canada, Sweden, and the UK, overtime working hours are limited to 48 h per week, including legal working hours, with Sweden averaging 7 days and the UK averaging 17 weeks. In France and Germany, employees can work up to 10 h per day, including legal working hours, within 48 h per week and 44 h per week on average for 12 weeks in France, and within 10 h per day on average for 24 weeks in Germany. Japan allows for overtime of up to 45 h per month and up to 360 h per year. According to OECD statistics, the proportion of employees working more than 49 h per week ranged from 5.9 to 18.3%, with Japan (18.3%) and the United States (14.2%) having the highest. Surcharges for overtime work are covered in Canada, France, Japan, and the USA. In Germany and Sweden, the surcharges may be allowed through a labor-management agreement. The UK has no such provision. With the exception of the USA, rest periods, holidays, and annual leave are legislated in all of the studied countries. In particular, annual leave is allowed for up to 20–30 days per year, depending on the length of service in a company. In France, there is an obligation of taking leaves of 12–24 consecutive working days between May 1 and October 31 every year, and similar provisions exist in Germany and Sweden. There is a system that allows unused leave to be carried over to the following year or later in some countries. It is allowed, if the extension is justified in Germany, for a one-year extension in Japan and five years in Sweden in case of leaves exceeding 20 days. In addition, Sweden allows companies to buy back 25 or more days of leave if they do not use it. As for other working conditions for general workers, flexible working time systems are allowed in France, Germany, Japan, the UK, and the USA under certain conditions, and restrictions on late-night work are in place in France, Japan, Sweden, and the UK.

### 3.2. LTC Support Systems for Employees Who Take Care of Family Members and Relatives, by Country

Table 3 depicts the support systems for employees related to LTC among seven countries [8,11,13,15,16,28,31,35,36,38,39,40,41,42]. It included laws related to LTC leave and LTC insurance system, and LTC leaves and allowance designated for employees. We summarized the current situations of LTC support systems by country below.

#### 3.2.1. Canada

In Canada, under the Employment Insurance Act, compassionate care leave is available for up to 28 weeks per year for workers with a dying family member, and up to 17 weeks for workers with a family member with a serious medical condition. For workers with family members with serious medical conditions, there is a form of leave related to critical illness of up to 17 weeks (up to 37 weeks for children under 18). Since both are unpaid leaves, Caregiving Benefits and Family Caregiver benefits have been established to provide financial support to workers. Under the former, those who have worked at least 600 h in the past 52 weeks and whose income has been reduced by at least 40% per week due to the terminal care of a family member such as a child, parent, or sibling, are entitled to one week of compassionate care benefits. After a waiting period, 55% of the average weekly wage for the insured period (up to $547 per week) will be paid for up to 26 weeks. The latter benefit provides up to 15 weeks of leave to care for a seriously ill family member (up to 35 weeks if the seriously ill family member is under the age of 18). In both cases, full-time work is not permitted while receiving the benefits.

#### 3.2.2. France

In France, the labor law stipulates the content of the current system. In addition to the conventional system of leave for relatives, there is a system of leave for close relatives (Congé de procheaidant) that includes those who regularly and frequently assist in daily life. There are no benefits associated with nursing care leave for employees, but the employer may not refuse the request for leave and must guarantee the same position after returning to work as before the period of leave. There is also a family solidarity leave (Congé de solidarité familiale) of up to three months to care for a terminally ill relative, which can be renewed once. The annual leave donation system has been brought into effect recently. It is the one that, upon agreement with the employer, a colleague in the same company can anonymously donate unused paid leave to a worker who has a seriously ill or disabled person needing care. If this system is applied, the worker’s salary is maintained during the leave period, and the leave period is calculated as actual working hours to determine the length of service. In addition, seniors aged 60 and above are entitled to receive self-help benefits for the elderly, including home and institutional services.

#### 3.2.3. Germany

In Germany, the Nursing Care Hours Act (Pflegezeitgesetz), enacted in 2008, the Family Care Hours Act (Familienpflegezeitgesetz), enacted in 2012, and the Law for a Better Harmonization of Family, Nursing Care and Work (Gesetz zur besseren Vereinbarkeit von Familie, Pflege und Beruf) guarantees workers’ rights. For example, in the case of an urgent need to provide nursing care, workers can use the short-term leave system for up to 10 days, and 90% of their previous wage (take-home pay) (with an upper limit) is paid as a nursing-care support allowance. In addition, in companies with 16 or more employees, they can take full or partial leave of absence for up to six months as part of the nursing care time system, and companies with 26 or more employees that require longer-term care can request a reduction in working hours to a minimum of 15 h per week and a maximum of 24 months (family care time system). These care hour programs are unpaid but interest-free loan programs are available. Demands of respite among informal caregivers have yet to be achieved with legal guidelines.

#### 3.2.4. Japan

In Japan, the Child Care and Family Care Leave Law stipulates a leave system and benefits for nursing care. The law guarantees the right to take a total of three nursing care leave days within a total of 93 days per family member, and also allows nursing care workers to take up to five days of nursing care leave per year (10 days per year for two or more family members) in one-day or half-day units. In addition, if the worker providing nursing care makes a request, overtime work can be restricted, with restrictions on overtime work exceeding 24 h per month and 150 h per year, as well as restrictions on late-night work between 10:00 p.m. and 5:00 a.m. Additionally, for workers who provide nursing care as a measure for shorter working hours, they are obliged to take one of the following measures, which can be used at least twice in three years: (1) shorter working hours system, (2) flextime system, (3) earlier or later start and end times, and (4) measures to assist with nursing care expenses.

#### 3.2.5. Sweden

In Sweden, the law on family care leave (Lagomledighet för närståendevård, 1988:1465) provides up to 100 days of leave per caregiver as family care (end-of-life care) leave. This system allows up to 100 days of leave per family member to be taken as family care (end-of-life care) leave, although the leave cannot be taken by more than one person at the same time. During the leave, a family care (end-of-life care) allowance is provided.

#### 3.2.6. The UK

In the UK, the Work and Families Act, The Care Act, and The Equal Act provide for care, but there is no system for care leave covering a period of longer than one or two months. To compensate for this, a time-off system for family members in emergencies and a flexible working system is applied, which allows employees to be excused from work or change their working conditions for a reasonable period without an expiration date. In such cases, caregiver allowances and national insurance caregiver exemptions can be used as salary guarantees, although there are income restrictions and other requirements for receiving such benefits.

#### 3.2.7. The USA

The Family and Medical Leave Act (FMLA) offers up to 12 weeks of unpaid leave in 12 months for childbirth, childcare, family nursing/LTC, and personal medical treatment. FLMA protects employees’ jobs, but there are several limitations such as no payment during leaves and organizational eligibility requirements. Its compensations are critical and several states including California and New York are implementing, at the state level, paid family leave. The Older Americans Act is a federal law that provides subsidies for long-term care services that do not fall under the category of medical care, but its budget is extremely small and there is basically no public long-term care insurance system. Medicare, which is included in the medical care category, and Medicaid, for those who can no longer afford to pay for their care, are the only means of coverage.

## 4. Discussion

In this study, we analyzed and evaluated the different LTC systems for employees engaged in informal caregiving in seven countries. While all countries must manage with an aging society involving the decline in the working-age population, informal care by employees is still a major mode of LTC for family members and close relatives. This is due to the fact that informal care usually targets people who are close to the employees and is perceived as a cost-effective method, as well as the high cost of the service provided by care facilities which are often insufficient in terms of both quantity and quality [2,3]. In addition, the socio-cultural norms in the regions lead women to engage in caregiving more often, and therefore female workers tend to limit their work and dedicate themselves to informal care [11]. However, the occupational health regulations and standards regarding LTC are not necessarily as comprehensive as those of childcare and are still being revised in response to changes in social conditions.

By conducting an extensive policy review, it has been found that occupational supports for employees involved in LTC as informal caregivers are relatively progressive in several countries. After several region-wide discussions and legislative amendments, these countries have established a unique system tailored to national circumstances. Particularly, it is notable that there is a system to effectively utilize mutual aid among employees. In France, unused paid leave by colleagues in a company can be donated to workers anonymously for LTC and seriously ill cases, upon agreement with the employer [35]. Although paid leave is not necessarily taken for the sole purpose of LTC, it is one of the most useful and practical means for employees engaged in LTC. In the USA, a maximum of 12-week unpaid leave is guaranteed by the federal Family and Medical Leave Act, but the Paid Family Leave system is being implemented at the state level [13,16,27]. On the other hand, many countries have set time limits for using paid leave. Although there are no international statistics available on annual leave usage, it can be assumed that there is a certain level of unused leave among employees. In Japan, the annual leave usage rate per worker in 2020 was 56.6%, and nearly half of leave remained unused [43]. This figure is the highest ever recorded since the survey began, and a lot of unused leave is abandoned since the carryover of the unused leave is only one year. There are reports that it is difficult to take leave due to socio-cultural norms and an unfavorable atmosphere [13]. In the USA, a large number of people are unaware of the state-based paid leave program itself [13,27]. It is, therefore, essential to establish a solid legal system for both workers and employers to foster a healthy working environment as an occupational health measure.

Recently, a discussion on how far to extend the number of care recipients covered by employees as informal care has been in motion. Until now, the range of informal care provided by employees has been considered as only to family members and relatives in many countries. In Sweden, however, there is a system in which the eligibility for LTC leave is not limited to family members or close relatives. Sweden has the lowest number of people per family among OECD countries, at 1.80 in 2015 [44]. The average number of family members in other countries is also declining, especially in urban areas, where this number is smaller than in rural areas. Additionally, it is not always the case that caregivers live near the family and relatives who are in need of LTC [29]. Under these circumstances, the entire community should protect those who need care, regardless of whether they are family members or not. In addition, from the perspective of improving the working environment for employees who take on the role of caregivers before and/or after work, there is a need to develop systems that allow them to continue to work flexibly while they can provide LTC when needed, as with childbirth and childcare-related systems. For example, in the UK, a system that allows employees to leave work for an appropriate period in case of an emergency, not limited to LTC care, and a flextime system have been established [38]. Although there is no current LTC leave system for 1–2 months in the UK, discrimination and harassment due to LTC are prohibited [38]. In Japan, labor regulations for employees who require LTC are mentioned in the same law as for childbirth and childcare, and other than temporary leave limited to LTC, there are the same provisions as normal working conditions such as shorter working hours, prohibition of late-night work, etc. [6]. Unlike childbirth and childcare, however, it is often unpredictable when LTC will occur due to types of illnesses such as cerebrovascular diseases; therefore it is not easy to make timely preparations for care in advance. For this reason, each country should continue to develop and flexibly implement relevant laws and regulations over the next 20 to 30 years so that employees can deal with LTC issues without being disadvantaged.

On the other hand, salary compensation and financial support for LTC-related leave are limited in all countries. A public survey revealed that more than half of respondents mentioned earning money as the main purpose of work, followed by finding a purpose in life or fulfilling one’s duties as a member of society [45]. In terms of the ideal job, many people cited a stable income, and many also wanted a job in which they were able to maintain their work–life balance [45]. In other words, the greatest threat is the negative impact on their daily lives financially so that they desire to avoid the loss of income due to LTC as much as possible. For this reason, it is necessary to enhance the safety net as a society, not only by the efforts of one company. In some countries, such systems are already in place. For example, in France, deductions for medical expenses are permitted, and in Germany, interest-free loans are available. In Japan, there is a system to compensate two-thirds of the salary for a certain period from employment insurance for those who take LTC-related leaves. Financial security is also of great concern in the USA, where only unpaid leaves are guaranteed under federal law, so the state-level supports are currently under-implemented in several states [27]. In Canada, also, tax-relief programs for informal caregivers are available at the state level, but they are usually scarcely known of and do little to support low-income caregivers [15]. Therefore, since LTC-related leaves and partial leaves are often unpaid in many countries, there is a need to further improve the system so that employees can engage in LTC without such stress.

Lastly, but not least, the health issues of the employees themselves who provide informal care are critical from the perspective of occupational health. The importance of respite care as protection for informal caregivers has been discussed elsewhere [8,20,23,24,25,26,27]. However, our study revealed that no country has legislated self-care for workers who are responsible for informal care. In the field of occupational health, health disorders are mostly related to compensation for illness and accidents during work and do not include matters outside of working hours. However, informal care is provided outside of working hours, and many health problems have been reported as a result of engaging in informal care, which may affect regular work [22]. These issues are more complex to resolve due to the need to consider one’s socioeconomic factors [16,17]. Protecting the health of employees as an occupational health policy will lead to corporate profits and social development, and protect a healthy work–life balance among them. Therefore, it would be valuable if respite care for employees who are engaged in LTC as informal caregivers were provided by each company or legislated for by the national government.

A limitation of the study is that it was difficult to provide quantitative data on the actual implementation of LTC-related leave and absence systems in the selected countries. This is due to the fact that there are almost no statistics available for international comparisons. Although data are released at the state and/or national levels [16,46,47], the level of data is far comparable at this moment. Only a few studies have shown such results [48]. Thus, even if an innovative system is proposed, it is not possible to evaluate the extent to which it is being utilized at this moment. However, in order to monitor and evaluate the impact of each system, such statistics should be regularly reported in every country and internationally comparable for a better implementation of the systems. 

## 5. Conclusions

In this study, we overviewed the LTC-related social support policies and systems for employees serving as informal caregivers in seven countries as part of occupational health measures. As a result, we found that various systems are in operation, taking into account the circumstances of each country. As the working-age population decreases, a system that incorporates the public assistance mechanism of providing unused paid leave to those in need will provide facilitate the more flexible operation of the existing occupational health system. Expanding the eligibility for LTC leave to non-family members will also be an asset for both employees and local communities in need of nursing care. In contrast, the health management of employees as informal caregivers is critical as part of occupational health measures, but progress made in this area is relatively scarce. It is strongly recommended that legislation and related programs be put in place to secure employee health conditions. Likewise, salary compensation and financial support for LTC-related leave should be better strengthened. Finally, it is essential to monitor and evaluate the progress and achievement of current legal systems and programs related to the social support of LTC among employees. So far, it has been difficult to quantify the extent to which employees engaged in informal caregiving benefited from the current policies and regulations as occupational health measures. These gaps should be further researched.

## Figures and Tables

**Table 1:** Socio-demographic indicators among seven countries.

	Population (× 1,000,000) ^1^	Working Age (15–64 Years Old) Population (%) ^1^		Elderly (65+ Years Old) Population (%) ^1^		Old Age Dependency Ratio ^1^		Life Expectancy (LE) at Birth, Both Sex (Years) ^2^	Healthy Life Expectancy (HALE) at Birth, Both Sex (Years) ^3^	Difference between LE and HALE
Year	2020	2020	Difference from 2005	2020	Difference from 2005	2020	2040	2019	2019	2019
**Canada**	38.01	66.1	Δ 3.2	18.0	5.5	0.274	0.384	82.2	71.3	10.9
**France**	67.35	61.6	Δ 3.5	20.6	4.3	0.335	0.468	82.5	72.1	10.4
**Germany**	83.16	64.4	Δ 2.4	21.9	3.0	0.340	0.477	81.7	70.9	10.8
**Japan**	125,71	59.3	Δ 6.8	28.8	8.6	0.489	0.656	84.3	74.1	10.2
**Sweden**	10.35	62.2	Δ 3.1	20.1	2.8	0.323	0.384	82.4	71.9	10.5
**UK**	67.08	63.5	Δ 2.5	18.6	2.7	0.294	0.398	81.4	70.1	11.3
**USA**	329.48	64.8	Δ 2.3	16.9	4.5	0.260	0.353	78.5	66.1	12.4

^1^ OECD Statistics: Demography and Population > Historical Population, at https://stats.oecd.org/Index.aspx?DataSetCode=HISTPOP, accessed on 26 November 2021. ^2^ WHO-GHO: Indicators > Life expectancy at birth (years), at https://www.who.int/data/gho/data/indicators/indicator-details/GHO/life-expectancy-at-birth-(years), accessed on 3 December 2021. ^3^ WHO-GHO: Themes > Topics > Indicator Groups > Healthy life expectancy (HALE), at https://www.who.int/data/gho/data/themes/topics/indicator-groups/indicator-group-details/GHO/healthy-life-expectancy-(hale), accessed on 3 December 2021.

**Table 2:** Occupational health regulations among seven countries.

Country [References]	Legal Working Hours and Average Hours Per Week Per Employed Person (2019–2020)	Overtime Work Hours, Wage, and % Employed Working 49 and More Hours Per Week (2019–2020)	Rest, Holidays and Special Leaves	Other Labor Standards
Canada [33,34,35]	8 h/day, 40 h/week (Labour Code), except Ontario State of 44 h/week ※Federal law (Labor Code) applies only to those who work across states (6% of the country, 900,000 people), and the rest are subject to state law Average hours per week per employed person: 32.1	48 h/week, including legal working hours 50% extra wage % employed working 49 and more hours per week: 10.6	Holidays: 1 day/week (Federal Law) Paid leave: 2–4 weeks (depends on employment period) Personal leave: Up to 5 days/year (first 3 days to be paid for those employed for more than 3 months)	No retirement system Variable working hours system
France [33,35]	35 h/week or 1607 h/year (Labor Act L3121-10) Average hours per week per employed person: 33.9	Up to 10 h/day, 48 h/week, and 44 h/week (12-week average), including legal working hours 25% extra wage or 75 min compensation break up to 43 h/week, or 50% or 90 min break for 43 h/week or more Up to 220 overtime hours/year, and 50–100% compensation rest for 220 h or more % employed working 49 and more hours per week: 9.1	Rest: At least 11 h between 2 working days Holidays: Sundays, not allowed to work 6 and more days/week, at least a 24-h weekly holidays, 25% extra wages if working on holidays Paid leave: 30 working days/year, with an obligation to take 12 to 24 consecutive working days as the main leave between 1 May–31 October	Variable working hours system: In a unit of 1–3 years, with a labor-management agreement Midnight work hours (definition): At least 9 consecutive hours including midnight-5 a.m. during 9 p.m.–7 a.m. with a labor-management or collective agreement, or all labors between 9 p.m.–6 a.m. without no definition of work time in the agreements Midnight work: Up to 8 h/day and 40 h/week, prohibited among adolescents younger than 18 years old
Germany [33,35]	8 h/day (Labor Time Act (Arbeitszeitgesetz)) Average hours per week per employed person: 34.2	Up to 10 h/day including legal working hours of 8 h/day (24-week average) Allowing 10 or more hours/day for a duty involving considerable waiting, with a labor agreement (even in that case, up to 48 h/week (12-month average)) No legal provisions regarding extra wages, but they may be stipulated in collective agreements % employed working 49 and more hours per week: 5.9	Rest: 11 consecutive hours or more after the end of each working day Holidays: Sundays and legal holidays (with exceptions), with no legal provisions regarding extra wages Paid leave: 24 days or more per calendar year among those employed more than 6 months (Federal Leave Act (Bundrsurlaubsgesetz)), including 12 consecutive days leave (can apply a different scheme under individual labor agreement), allowing carry-over to the next year only if justified	Variable working hours system: In a unit of 6 months or 24 weeks
Japan [33]	8 h/day, 40 h/week (Labor Standards Act) Average hours per week per employed person: 37.8	Up to 45 h/week, 360 h/year (36 labor agreement) 25% or more extra wage for overtime beyond scheduled working hours % employed working 49 and more hours per week: 18.3	Holidays: 1 or more day/week, 4 or more days/4 weeks, 35% or more extra wage if working on holidays Paid leave: 10 or more days/6 months, up to 20 days/year; possible to carry over to the next year	Variable working hours system: In a unit of 1 week, month, or year, within the legal working hours Midnight work (10 p.m.-5 a.m.): 25% or more extra wage
Sweden [31,36]	40 h/week (Labor Time Act (Arbetstidslag) (1982:673)) Average hours per week per employed person: 34.9	Average of 48 h or less per every 7 days, including legal working hours General overtime (allmän övertid): Up to 48 h in 4 weeks (50 h in calendar month), within 200 h per calendar year Extra overtime (extra övertid): Up to 150 h per calendar year in addition to regular overtime under special circumstances, with a maximum of 48 h in 4 weeks (50 h in calendar month) No legal provisions regarding extra wages or flexible working hours system, and rules are stipulated in collective agreements % employed working 49 and more hours per week: 5.7	Rest: 11 consecutive hours or more in every 24 h including midnight-5 am, and 36 consecutive hours or more in every 7 days possibly including a weekend Break time: At least 5 consecutive hours Paid leave: 25 days/year (Annual Leave Act (Semesterlag (1977:480) with allowance (semesterlön); Of these, 4 weeks can be taken consecutively between June-August; Annual paid leave exceeding 20 days can be carried over from the following year up to 5 years later; A leave allowance will be paid for the unused leaves carried over for more than 25 days.	Night work: Up to 8 h in average per 24 h within the last 4 reference months among those who normally work at 10 p.m.-6 a.m. for 3 h or more of the daily working hours or one-third or more of the annual working hours; not exceed 8 h every 24 h when engaging in labor with a special danger and heavy physical and mental burden
UK [33,35]	Up to 48 h/week (17-week average), including overtime work hours (Labor Time Regulation) Average hours per week per employed person: 35.9	No legal provisions regarding extra wages for overtime work % employed working 49 and more hours per week: 11.4	Rest: At least 11 consecutive hours per any 24 h, 24 consecutive hours or more per week, or 48 consecutive hours or more per 2 weeks Break time: A minimum of 20 min break if the daily working hours are 6 h or more Holidays: 1 day/week (2 days/week for young workers), no regulation about the extra wage for holiday work Paid leave: working day/week x 5.6 days (up to 28 days/year); can take leaves since the first day of working	Midnight work: Up to 8 h/day in 17 reference weeks; Up to 8 h per 24 h for labor with a special danger or heavy physical and mental burden; Free annual health check-up (thru questionnaire) including before employment Flexible Working: Rights of job sharing, working from home, part-time work, compressed hours of weekly work, flextime for those engaging in employment for more than 26 consecutive weeks
USA [33,35,37]	40 h/week under federal rule (Fair Labor Standards Act of 1938: FLSA) 40 h/week in more than a half of states, additionally 8 h/day in the states of Alaska and California Average hours per week per employed person: 35.9	No federal law to set an upper limit on working hours 50% extra wage for work over 40 h/week under the federal system % employed working 49 and more hours per week: 14.2	No federal law mentioning rest, holidays, and annual paid leaves No laws and ordinances on extra wage for holiday work	Variable working hours system: In a unit of 26 or 52 weeks with a collective agreement No federal law regarding midnight work

**Table 3:** Supports for employees of those in need of nursing or long-term care (LTC) in seven countries.

Country [References]	Law Related to LTC Leave and LTC Insurance System	LTC Leave and Allowance for Employees
Canada [15,35]	Employment Insurance Act	Compassionate care leave: Up to 28 weeks of unpaid leave per year among employees with families near death, with a medical certificate Leave related to critical illness: Up to 17 weeks of unpaid leave among employees with families with serious medical conditions (up to 37 weeks for children under the age of 18) Caregiving Benefits: 55% of the average weekly wage for the insured period (up to $ 547/week) as a compassionate care benefit up to 26 weeks after a one-week waiting period for employees who worked for 600 h or more covered by employment insurance in the past 52 weeks with a decreased salary of 40% or more per week due to nursing care for end-of-life family members Family Caregiver Benefit: Up to 15 weeks if taking leave to care for a seriously ill family member (up to 35 weeks for those under the age of 18) ※In either case, full-time work is not permitted while receiving the benefits. Tax relief programs available at the state level
France [11,28,35]	Labor Code (Code du Travail) Act on adapting society to an aging population Personal allowance for autonomy (60 years and more) (Allocation personnalisée d’autonomie)	Family support leave (Congé de soutien familial, 2007), Caregiver leave (Congé de proche aidant, 2017): 3 months (up to 1 year) among those working more than 2 years; Salary to be paid or not by the employer’s decision; No relevant allowance but pension reserves and medical insurance premium to be covered by the government; possible to switch to part-time work or to take leaves in parts instead of taking full leaves Family solidarity leave (Congé de solidarité familiale): Up to 3 months to care for end-of-life relatives (1-time renewable) A system in which a colleague gives unused paid leaves anonymously and gratuitously to a worker who has a serious illness or disability who needs long-term care (need an agreement with the employer): Recipient’s salary is maintained during the leave, which is included in the actual working hours for calculating the length of service
Germany [8,11,35,39,40]	Caregiver Leave Act (Pflegezeitgesetz, 2008) Family Caregiver Leave Act (Familienpflegezeitgesetz, 2012) Act to Improve the Reconciliation of Family, Care and Work (Gesetz zur besseren Vereinbarkeit von Familie, Pflege und Beruf, 2015) LTC Insurance (Pflegeversicherung) Long Term Care Strengthening Act (PSG I & II)	Short-term care leave: Up to 10 days in case of emergent nursing care, regardless of company size; Nursing care support allowance of 90% net income (with upper limit) Statutory right to the 6 months’ care leave: full- or part-time absence for employees in companies with more than 15 workers; Available for an interest-free loan system Statutory right to work part-time: Up to 24 months for employees in companies with more than 25 workers Family care time: Reduction of a working hour by a minimum of 15 h up to 24 months, including 6 months’ time-off work; Available for an interest-free loan system
Japan [42]	Act on Childcare Leave, Nursing Care Leave, and Other Measures for the Welfare of Workers Caring for Children or Other Family Members (Childcare and Nursing Care Leave Act) LTC insurance supported by Nursing Care Insurance Act	(Temporary) absence from work due to nursing care: Up to 3 times within a total of 93 days per family member Nursing care leave: Up to 5 days/year/family member (10 days/year/2 or more family members) Restriction on working in excess of scheduled working hours upon request by employees Overtime work: Up to 24 h/month, 150 h/year No midnight work between 10 pm-5 am upon request by employees Reducing scheduled working hours system including flextime and staggered working hours, which are available at least twice in three years
Sweden [31,36,41]	Legal leave for the care of close relatives (Lagomledighet för närståendevård (1988:1465)) Social Service Act Attendance allowance (hemvårdsbidrag)	Family care (end-of-life care) leave: Up to 100 days for a person requiring LTC, regardless of residential status (home or facility); No multiple employees can take leaves for the same person requiring LTC but caregivers are not limited to family members Related party benefit (Närståendepenning) during the above leave Carer allowance (anhöriganställning): that municipality employs a family member under 65 years of age to do the care work and gives the same salary and similar social security as for home-help workers in the municipality’s own services
UK [38]	Work and Families Act 2006 The Care Act 2014 The Equal Act 2010	No LTC leave system assuming 1–2 months or more Carers Allowance: £62.1/week per caregiver, with eligibility requirements such as a weekly income of less than £110 Carer’s Credit exemption for national insurance, with eligibility requirements Time-off in case of emergency: Employees can leave work for a reasonable length of time Flexible work system: Changes in working conditions, including part-time job, flextime, job sharing Prohibition of direct discrimination and harassment due to being a caregiver
USA [13,16,35]	Family and Medical Leave Act (FMLA) Older Americans Act ※But no public LTC insurance system (only covered by Medicare included in medical care and Medicaid when self-pay becomes impossible)	Up to 12 weeks of unpaid leave in 12 months for childbirth, childcare, family nursing/LTC, and personal medical treatment (conditions may apply) (FLMA) State-based paid family leave programs available, such as "temporary caregiver insurance" or "family leave insurance" State-based paid sick time legislation available Subsidies for LTC services that do not fall into the medical category under the U.S. Elderly Act (budget is extremely small)

## Data Availability

The data presented in this study and references are available in Table 1, Table 2 and Table 3.
